# Quantification of breast lymphoedema following conservative breast cancer treatment: a systematic review

**DOI:** 10.1007/s11764-022-01278-w

**Published:** 2022-10-27

**Authors:** Nicola Fearn, Catalina Llanos, Elizabeth Dylke, Kirsty Stuart, Sharon Kilbreath

**Affiliations:** 1 Sydney School of Health Sciences, Faculty of Medicine and Health, The University of Sydney, Susan Wakil Health Sciences Building, Western Avenue, Camperdown, NSW Australia; 2 Westmead Breast Cancer Institute, Westmead Hospital, Sydney, NSW Australia; 3 Sydney Medical School, The University of Sydney, Sydney, NSW Australia; 4 Department of Radiation Oncology, Crown Princess Mary Cancer Centre, Westmead Hospital, Sydney, NSW Australia

**Keywords:** Assessment, Measurement properties, COSMIN, Breast lymphoedema, Breast conserving surgery, Breast cancer

## Abstract

**Purpose:**

Breast lymphoedema is a possible side effect of breast conserving surgery, but it is poorly understood. This is due, in part, to difficulty assessing the breast. This systematic review described outcome measures that quantify breast lymphoedema signs and symptoms and evaluated the measurement properties for these outcome measures.

**Method:**

Seven databases were searched using terms in four categories: breast cancer, lymphoedema and oedema, clinician reported (ClinROM) and patient reported outcome measures (PROM) and psychometric and measurement properties. Two reviewers independently reviewed studies and completed quality assessments. The Consensus-based Standards for the Selection of Health Measurement Instruments (COSMIN) methodology was used for studies including measurement property evidence.

**Results:**

Fifty-six papers were included with thirteen questionnaires, eight patient-reported rating scales, seven physical measures, seven clinician-rating scales and four imaging techniques used to quantify breast lymphoedema. Based on COSMIN methodology, one ClinROM had sufficient reliability, ultrasound measuring dermal thickness. Tissue dielectric constant (TDC) measuring local tissue water had promising reliability. Four questionnaires had sufficient content validity (BLYSS, BLSQ, BrEQ and LYMQOL-Breast).

**Conclusions:**

Ultrasound is recommended to reliably assess breast lymphoedema signs. No PROM can be recommended with confidence, but BLYSS, BLSQ, BrEQ and LYMQOL-Breast are promising. Further research is recommended to improve evidence of measurement properties for outcome measures.

**Implications for Cancer Survivors:**

There are many approaches to assess breast lymphoedema, but currently, only ultrasound can be recommended for use, with others, such as TDC and questionnaires, showing promise. Further research is required for all approaches to improve evidence of measurement properties.

## Introduction

Breast conserving surgery with adjuvant radiotherapy is a common treatment regimen for women with early breast cancer as it leads to better quality of life [1] and improved survival to that of women undergoing mastectomy [2, 3]. Unfortunately, breast lymphoedema can be a painful and distressing complication of the breast conserving treatment regime [4, 5]. Breast lymphoedema is not well understood and poorly addressed by health professionals [6]. The reported incidence of breast lymphoedema varies considerably across studies, ranging from 0 to 90% due to variances in the definition and tools selected to diagnose and quantify breast lymphoedema [7, 8].

Assessments of lymphoedema in the limbs have been validated [9–13]; however, it is unknown if those tools can be used in the assessment of breast lymphoedema. Measurement of lymphoedema in the breast differs to that in the arm as the breast is the direct recipient of the surgical and radiotherapy treatment. These treatments change the volume and tissue architecture of the affected breast, reducing the usefulness of measuring the breast pre-operatively or measuring the contralateral breast as a direct comparator. Changes to the breast caused by surgery and radiotherapy may also make it more difficult to distinguish between treatment impacts and those changes caused by presence of breast lymphoedema. Furthermore, self-reported questionnaires for lymphoedema have tended to focus on and be tested with people with limb lymphoedema rather than on populations with breast or midline lymphoedema [11, 13].

This systematic review describes what outcome measures are available to quantify breast lymphoedema signs and symptoms following breast conserving surgery and evaluates the evidence underpinning the measurement properties for these assessment tools or approaches, where available.

## Methods

The systematic review was registered with the International Prospective Register of Systematic Reviews on 05 July 2020 (PROSPERO registration no: CRD42020183851).

The review was conducted according to the Preferred Reporting Items for Systematic Reviews and Meta-Analyses (PRISMA) guidelines [14] and Consensus-based Standards for the Selection of Health Measurement Instruments (COSMIN) guideline for systematic reviews [15–17].

### Database search

Five electronic databases were searched including Medline, Embase, CINAHL, Web of Science and Scopus as well as Trove and ProQuest Dissertations & Theses Global for theses that explored breast lymphoedema measurement. Searches were conducted with support from a librarian at the University of Sydney. Search terms were grouped into four categories relating to (i) breast cancer; (ii) lymphoedema and oedema; (iii) clinician-reported (ClinROM) and patient-reported outcome measures (PROM); and (iv) psychometric and measurement properties. The full Medline search strategy is described in Online Resource 1. The initial search was conducted on 19th April 2020 and repeated on 19th August 2021 and 14th February 2022 to check for recently published articles. There was no restriction on date of publications, but only articles published in English were included.

### Selection criteria

Studies were included in which an assessment was used to quantify breast lymphoedema and related symptoms (e.g. peau d'orange, induration, hardness, heaviness, discomfort, skin redness) in adult women following breast conserving surgery (lumpectomy/wide local excision) for breast cancer. Women may have been treated with chemotherapy, radiotherapy and/or immunotherapy. Theses were included when publicly available online or provided by authors following request. Studies with men, women under 18 years old, women treated with mastectomy and/or reconstruction and assessment for lymphoedema in areas of the body other than the breast were excluded. Studies only using toxicity or cosmesis rating scales (e.g. CTCAE, LENT SOMA, National Cancer, Institute Canada-Common Toxicity Criteria 2, Harvard Breast Cosmesis Scale, Outcome by American Society for Radiation Oncology (ASTRO) Consensus Panel (CP) group and acute and late RTOG scales) were also excluded.

### Study selection

Duplicates were removed using electronic and manual review in EndNOTE (version X9) with additional duplicates identified when titles were imported to Covidence systematic review software (Veritas Health Innovation, Melbourne, Australia (available at www.covidence.org). Titles and abstracts, followed by full text papers, were independently screened by two reviewers (NF, SK, CL). Reference lists of included full text papers were examined to identify additional appropriate studies. When disagreements on study eligibility occurred, consensus was reached through discussion as a team.

### Data extraction and analysis

Two reviewers independently extracted data (SK and NF or CL and NF) using Covidence data extraction template (version 1). Information extracted included study design, participant demographics, treatment history and the stage at which the assessments took place in the participants’ cancer treatment timeline (e.g. time since diagnosis, surgery and/or radiotherapy). The purpose of the assessment (e.g. assessing treatment side effects, quality of life or measuring outcomes from an intervention to treat breast lymphoedema) and details pertaining to the measurement properties of the tools were also extracted where available. If there were missing data or data from participants following breast conserving surgery or breast lymphoedema were not presented separately, authors were contacted requesting this data.

### Quality assessment

Several tools were used to assess the quality and risk of bias of included papers due to the variety of study designs included in this review. All assessments were completed by two reviewers (NF, SK, CL) independently and all disagreements were resolved through discussion until agreement was made. Included papers that had been authored by SK were assessed by other team members (NF and CL) to prevent potential bias in quality assessment.

The Cochrane tool for assessing risk of bias (RoB) in randomised trials, version 2 (RoB 2) [18], was used for the randomised controlled trials, and the quality assessment for cohort or non-randomised experimental studies was completed using the National Heart, Lung and Blood institute (NHLBI) Quality Assessment Tool for before-after (pre-post) studies with no control group (URL: www.nhlbi.nih.gov/health-topics/study-quality-assessment-tools (accessed 26 October 2020).

Consensus-based Standards for the Selection of Health Measurement Instruments (COSMIN) Risk of Bias checklist adapted for clinical measures (ClinROMs) [15] was completed for studies including measurement properties for clinician rating scales, measurement device or imaging tool. The COSMIN ROB checklist for patient reported outcome measures (PROMs) [16] was used for studies including measurement properties for PROMs. The studies were assessed separately against each standard using the four-point scale (very good, adequate, doubtful or inadequate), and then quality was rated using the “worst-score-counts method” [17]. The quality of PROM development was evaluated first, followed by the quality of content validity studies, and these results were combined to rate the content validity overall based on relevance, comprehensiveness and comprehensibility for breast lymphoedema measurement in women following conservative breast cancer treatment. Next, the other eight measurement properties were evaluated. Finally, the overall quality of evidence for each tool was graded using the modified GRADE (Grading of Recommendations, Assessment, Development and Evaluation) approach incorporating the assessment of risk of bias, inconsistency, imprecision and indirectness to grade the quality of evidence as high, moderate, low or very low quality [17].

Recommendations for the use of tools or approaches were categorised, based on the evidence, as (A) recommended (PROM, evidence of sufficient content validity and internal consistency; ClinROM, evidence of sufficient face validity and reliability), (B) promising (additional validation studies required, not categorised as A or C) or (C) insufficient (high quality evidence of insufficient measurement property) [17].

### Data synthesis

A narrative synthesis of the findings from the included studies was performed for the breast lymphoedema measurement tools or approaches and available measurement properties. Meta-analysis was not conducted as the review was of the assessment tools, not treatment outcomes or efficacy of treatment.

## Results

**Fig. 1:**
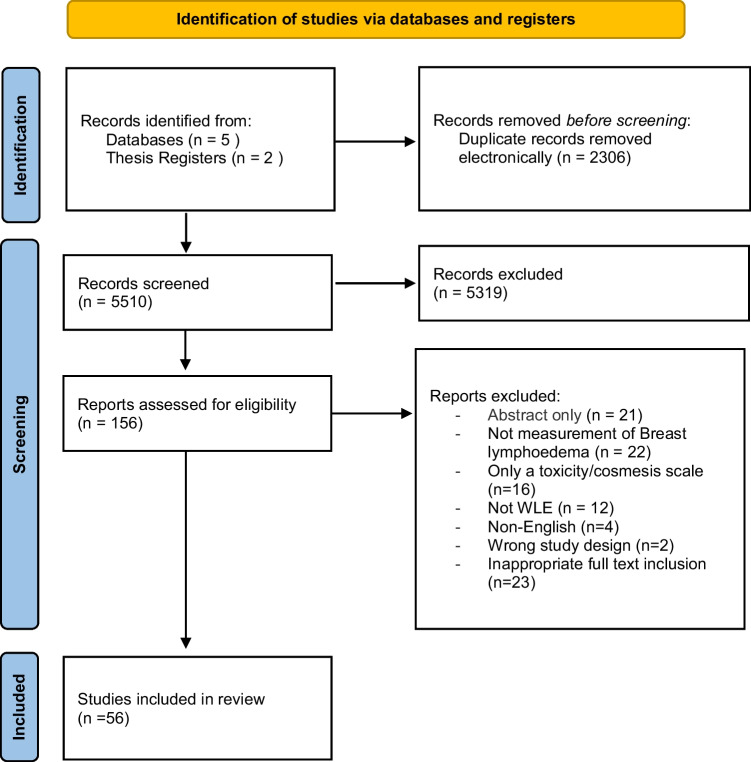
Preferred Reporting Items for Systematic Reviews and Meta-Analyses (PRISMA) diagram [14]

The search of databases identified 7805 papers and 169 theses titles, with 2306 duplicates removed. Following title and abstract review, 156 papers progressed to full paper review. Fifty-four papers and two theses met the inclusion criteria for this review (Fig. 1) following review of the full papers. Thirty-two studies measured breast lymphoedema signs and symptoms as a side effect of cancer treatments including breast conserving surgery and radiotherapy [19–50]. Six studies measured the outcome of specific breast lymphoedema interventions [51–56]. Seventeen studies reported on the measurement properties of the tools used and were further analysed with the COSMIN framework [31, 38, 57–71].

Fourteen studies used a combination of ClinROM and PROM, including all but one [52] of the breast lymphoedema interventions studies. ClinROMs alone were used in 23 studies, and PROMs alone in 20 studies. Most studies (62.5%) used at least two different tools, with one study using seven [59].

### Characteristics of clinician reported outcome measures

Signs of breast lymphoedema were quantified using multiple tools and approaches (Table 1). Breast tissue dermal thickness was measured using ultrasound (*n* = 12) [21–24, 35, 36, 54, 57–60, 72] and mammography (*n* = 3) [26–28]; local tissue water was measured using tissue dielectric constant (TDC) (*n* = 8) [19, 20, 51–53, 59, 61, 73]; breast volume was measured using three-dimensional surface imagery (3D-SI: *n* = 4) [37, 40, 56, 63], magnetic resonance imaging (MRI: *n* = 1) [41] and anthropomorphic techniques (*n* = 1) [36]; extracellular fluid volume was measured using bioimpedance spectroscopy (BIS: *n* = 3) [54, 62, 74]; tissue resistance was measured using tonometry (*n* = 3) [55, 59, 62], the pitting test (*n* = 2) [59, 61] and indentation force (*n* = 1) [52]; and dermal backflow/compensatory drainage pathways was visualised with indocyanine green imaging (ICG: *n* = 1) [73]. Additionally, clinician rating scales (*n* = 8) [29, 31, 33–35, 37, 47, 72] were used to identify presence of changes to the appearance, size or texture of the breast tissue. Rating scales were also used by clinicians to identify or grade indicators of breast lymphoedema seen using ultrasound or mammography, including signs of parenchymal or cutaneous oedema, trabecular thickening and skin elasticity (*n* = 8) [24–29, 35, 36].

**Table 1:** Characteristic of studies, assessment tools and populations, organised by assessment tool

Author/ tool *Sample size N* =	Assessment location/s *BLE duration*	Outcome assessed *Study purpose*	Age: mean ± SD (range) or Median (IQR)	Measurement timepoints *Times since surgery/RT*	NHLBI quality assessment/ ROB 2
Clinician reported outcome measures					
Ultrasound					
Kerrigan 2021 [72] *N* = *30*	Single site (6 o’clock)	Dermal thickness *BLE assessment*	Range ~ > 40 to > 70 years	Single measurement *6–24 months post-surgery*	NHLBI-Fair
Kilbreath 2021 [60] *N* = *88*	Quadrants *BLE* > *3 months*	Dermal thickness *Reliability, measurement error*	56.8 ± 9.6 years	Baseline and 12 weeks *11–26 months post-surgery*	NHLBI-Good
Riches 2020 [59] *N* = *40, retest n* = *25*	Quadrants	Dermal thickness *BLE assessment methods*	BLE = 59.98 ± 10.58 years All = 61.1 ± 9.6 years (29–80)	Baseline and 5–10 days later *6 months to 12 years post-surgery*	NHLBI-Fair
Kilbreath 2020 [54] *N* = *89*	Quadrants *BLE* > *3 months*	Dermal thickness *RCT: Intervention-exercise*	Control age: 59.5 ± 8.0 years Exercise age: 53.7 ± 10.4 years	Baseline and 12 weeks	ROB-Low
Verbelen 2020 [58] *N* = *55*	Quadrants	Dermal thickness *Validating questionnaire*	BLE: 58.2 ± 11.48 years No BLE: 63.0 ± 10.1 years	Two measures, 24–48 h apart	NHLBI-Good
Dylke 2018 [57] *N* = *38*	Quadrants *BLE* > *3 months*	Dermal thickness *Reliability study*	> 18 years	Single measurement	NHLBI-Fair
Garnier 2017 [21] *N* = *34*	Two sites	Dermal thickness *RT side effects*	61.5 [IQR 53.0–68.0] years	Single measurement, final RT treatment	NHLBI-Fair
Adriaenssens 2012 [36] *N* = *20*	Quadrants	Dermal thickness, elastography *RT side effects and BLE diagnosis*	58.9 ± 12.1 years	Pre-surgery and post RT	NHLBI-Fair
Wratten 2007 [24] *N* = *52*	Medial and lateral breast	Dermal thickness, imaging signs *RT side effects*	55 (31–74) years	Pre RT, weekly during RT, 2, 6 weeks, 4, 6, 12 and 24 months *post RT*	NHLBI-Fair
Della sala 2006 [25] *N* = *90*	Whole breast	Imaging signs *RT side effects*	IORT: 62 (45–79) years RT: 60 (40–78) years	Baseline, 12, 24 months *post RT*	NHLBI-Poor
Ronka 2004 [35] *N* = *160*	Quadrants and whole breast	Dermal thickness, imaging signs *Surgical side effects*	SNB: 59 (39–77) years AC: 58 (37–80) years No AC: 58 (39–81) years	Single measurement *Mean 12.6 (11.3–18.8) months post-surgery*	NHLBI-Fair
Wratten 2002 [22], 2000 [23] *N* = *11*	Medial and lateral breast	Dermal thickness *Surgical side effects*	BLE: 56.54 ± 11.1 (35–71) years	Single measurement	NHLBI-Fair
Tissue dielectric constant					
Johansson 2020 [51] *N* = *56*	Quadrants and mean	Local tissue water *RCT: Int—compression*	Comp = 61.9 ± 7.6 years No comp = 61.3 ± 9.6 years	3 months post RT and 9 months	ROB-Some concerns
Heydon-White 2020 [73] *N* = *10*	Quadrants	Local tissue water *Assessment of tools*	54 ± 15 (36–81)	Single measurement	NHLBI-Fair
De Vrieze 2019 [61] *N* = *9*	Single site *BLE 74* ± *44 months*	Local tissue water *Reliability, measurement error*	65 ± 8 years	Single measurement	NHLBI-Fair
Riches 2020 [59] *N* = *40, retest n* = *25*	Quadrants	Local tissue water *BLE assessment methods*	BLE = 59.98 ± 10.58 years All = 61.1 ± 9.6 years (29–80)	Baseline and 5–10 days later *6 months to 12 years post-surgery*	NHLBI-Fair
Collins 2018 [53] *N* = *14*	Quadrants and mean	Local tissue water *RCT: Int-Kinesiotape*	Age: Kinesio = 64.1 ± 5.9 years Usual = 53.9 ± 10.4 years	Baseline, EOT and 6 weeks post treatment *At least 4 weeks post radiotherapy*	ROB-Low
Mayrovitz 2017 [52] *N* = *12*	Single site	Local tissue water *Intervention–skin cooling*	61.0 ± 12.1 (38–88 years)	Single session, two measurements: pre and post cool	NHLBI-Poor
Johansson 2015 [19] *N* = *65*	Quadrants and mean	Local tissue water *RT side effects*	61.2 ± 8.1 years	Pre RT, 3, 6 months, 1, 2 years post RT	NHLBI-Good
Johansson 2014 [20] *N* = *118*	Quadrants & mean	Local tissue water *RT side effects*	61.3 ± 8.4 years	Seven measurements: Pre RT, weekly during RT, 2, 4 weeks post RT	NHLBI-Fair
Mammography					
Tian 2016 [26] *N* = *89*	Whole breast	Dermal thickness, imaging signs *RT Side effects*	Med 60 (33–83) years	Single measurement *Med 48 months post RT*	NHLBI-Fair
Carvalho 2011 [27] *N* = *60*	Whole breast	Dermal thickness, imaging signs *RT side effects*	ELIOT: 64.1 ± 8.9 years RT: 54.3 ± 8.8 years	Single measurement *12 months post RT*	NHLBI-Fair
Kuzmiak 2009 [28] *N* = *64*	Whole breast	Dermal thickness, imaging signs *RT side effects*	IORT: 70 (48–92) years WBRT: 62 (45–82) years	Single measurement *12 months post RT*	NHLBI-Poor
Della Sala 2006 [25] *N* = *90*	Whole breast	Imaging signs *RT side effects*	IORT: 62 (45–79) years RT: 60 (40–78) years	Baseline, 12, 24 months *post RT*	NHLBI-Poor
Vuorela 1989 [29] *N* = *14*	Whole breast	Imaging signs *RT side effects*	48.5 (30–75) years	Single measurement *1–10 months/12–41 months post RT*	NHLBI-Poor
Three-dimensional surface imagery					
Leusink 2021 [63] *N* = *31*	Whole breast	Breast volume *Assessment of tool*	NR	Single measurement *Between 1–6 years post BCT*	NHLBI-Fair
Koban 2020 [37] *N* = *38*	Whole breast	Breast volume *RT side effects*	Med 57 years (30–80 years)	Baseline, weeks 1, 2, 3, 4, 5, 6 and 7 and 3 months *post RT*	NHLBI-Poor
Chapman 2020 [40] *N* = *77*	Whole breast	Breast volume *RCT-RT side effects*	NR	Post-surgery/RT *3 years post RT*	ROB-Some concerns
Jahr 2008 [56] *N* = *21*	Whole breast	Breast volume *RCT- interventions MLD* ± *deep oscillation*	Treat: 56.6 (41–65) years Control: 62.0 (42–71) years	Three measurements: baseline, 4 weeks (end of intervention), 8 weeks post Intervention	ROB-Some concerns
Bioimpedance spectroscopy					
Ward 2020 [74] *N* = *41*	Whole breast-R_0_ ratio > *3 months*	Extracellular fluid *Assessment of BLE tool*	> 18 years	Single measurement	NHLBI-Good
Kilbreath 2020 [54] *N* = *89*	Whole breast-R_0_ ratio > *3 months*	Extracellular fluid *RCT: Intervention-exercise*	Control age: 59.5 ± 8.0 years Exercise age: 53.7 ± 10.4 years	Baseline and 12 weeks	ROB-Low
Moseley 2008 [62] *N* = *14*	Quadrants-R_0_ ratio	Extracellular fluid *Assessment of BLE tool*	61.6 ± 9.7 years	Single measurement *Mean 8.7* ± *4.7 years post-surgery*	NHLBI-Poor
Pitting test					
Riches 2020 [59] *N* = *40, retest n* = *25*	Quadrants	Tissue resistance *BLE assessment methods*	BLE:59.98 ± 10.58 years All: 61.1 ± 9.6 years (29–80)	Baseline and 5–10 days later *6 months to 12 years post-surgery*	NHLBI-Fair
De Vrieze 2019 [61] *N* = *9*	1 site *74 months* ± *44*	Tissue resistance *Reliability, measurement error*	65 ± 8 years	Single measurement	NHLBI-Fair
Tonometry					
Riches 2020 [59] *N* = *40, retest n* = *25*	Quadrants	Tissue resistance *BLE assessment methods*	BLE:59.98 ± 10.58 years All:61.1 ± 9.6 years (29–80)	Baseline and 5–10 days later *6 months to 12 years post-surgery*	NHLBI-Fair
Ashforth 2011 [55] *N* = *4*	Single site	Tissue resistance *Int: JoViPitPak, SLD, compression*	39–55 years	Baseline, 2 weeks, 5 weeks of intervention	NHLBI-Poor
Moseley 2008 [62] *N* = *14*	Quadrants	Tissue resistance *Assessment of BLE tool*	61.6 ± 9.7 years	Single measurement *Mean 8.7* ± *4.7 years post-surgery*	NHLBI-Poor
Indentation force					
Mayrovitz 2017 [52] *N* = *12*	Single site	Tissue resistance *Intervention- skin cooling*	61.0 ± 12.1 (38–88 years)	Single session, two measurements: pre and post cool	NHLBI-Poor
ICG					
Heydon-white 2020 [73] *N* = *10*	Whole breast	Lymphatic pathways/backflow *Assessment of tools*	54 ± 15 (36–81)	Single measurement	NHLBI-Fair
Anthropomorphic					
Adriaenssens 2012 [36] *N* = *20*	Whole breast	Breast volume (Qiao technique[81]) *RT side effects and BLE diagnosis*	58.9 ± 12.1 years	Pre-surgery and post RT	NHLBI-Fair
MRI					
Pukancsik 2017 [41] *N* = *200*	Whole breast	Breast volume	56 (32–70) years	Once, 12 months post-surgery	NHLBI-Poor
Clinical rating scales					
Kerrigan 2021 [72] *N* = *30*	Whole breast	Soft tissue *BLE assessment*	Range ~ > 40 to > 70 years	Single measurement *6–24 months post-surgery*	NHLBI-Fair
Ronka 2004 [35] *N* = *160*	Whole breast	Size, tenderness, pigmentation, skin condition *Surgical side effects*	SNB: 59 (39–77) years AC + : 58 (37–80) years AC-: 58 (39–81) years	Single measurement *12.6 (11.3–18.8) months post-surgery*	NHLBI-Fair
Koban 2020 [37] *N* = *38*	Whole breast	Skin erythema *RT side effects*	Med 57 years (30–80 years)	Baseline, weeks 1, 2, 3, 4, 5, 6 and 7 and 3 months *post RT*	NHLBI-Poor
Degnim 2012 [31] *N* = *124*	Quadrants, nipple, areolar	Oedema, erythema *Assessment of BLE tool*	Med 56.5 (36–85) years	1, 3, 6 and 12 months post op *Median follow up 11 months (3–14 months)*	NHLBI-Fair
Pezner 1985 [33] *N* = *45*	Whole breast	Breast size, peau d’orange, skin erythema, hyperpigmented pores *RT side effects*	55 (34–68)	Single measurement *18 months post RT (5–42 months)*	NHLBI-Poor
Clarke 1982 [34] *N* = *76*	Whole breast	Oedema, fibrosis, hyperpigmentation, thrombophlebitis *RT side effects, diagnosis of BLE*	Med 46 years	Multiple measurements *Follow-up Med 23 months (12 mths-7.5 years) from diagnosis*	NHLBI-Poor
Vuorela 1989 [29] *N* = *14*	Whole breast	Skin and breast consistency *RT side effects*	48.5 (30–75) years	Single measurement *1–10 months/ 12–41 months post RT*	NHLBI-Poor
Patient-reported outcome measures					
Author Sample size n =	Languages *BLE duration*	Construct/symptoms *Study purpose*	Age: mean ± SD (range)	Measurement timepoints *Times since surgery/RT*	NHLBI QA/ROB2
BrEQ					
Verbelen 2020 [58] *N* = *55*	Dutch (English version not validated)	Symptom experience *Validating questionnaire*	BLE: 58.2 ± 11.48 years No BLE: 63.0 ± 10.1 years	Two measures, 24–48 h apart	NHLBI-Good
BLYSS					
Smith 2013 [64] *N* = *50 (PROM develop),* *N* = *30 (PROM test)*	English *Develop: 0.2–9 years* *Test: 0.25–14 years*	Symptom experience *Validating questionnaire/reliability*	Develop: 62.5 ± 8.5 (45–85) years Testing: 46–82 years	Test: repeated BLYSS 24 h later *Develop: 5.4* ± *26 (1–11 years)* *Test: 0–21 years post-surgery*	NHLBI-Fair
LSIDS-Trunk					
Kilbreath 2020 [54] *N* = *89*	English *BLE* > *3 months*	Symptom experience: intensity and distress*. RCT: Intervention—exercise*	Control: 59.5 ± 8.0 years Exercise: 53.7 ± 10.4 years	Baseline and 12 weeks	ROB-Low
BLSQ					
Riches 2020 [59] *N* = *40, retest n* = *25*	English	Symptom experience *BLE assessment methods*	BLE: 59.98 ± 10.58 years All: 61.1 ± 9.6 years (29–80)	Baseline and 5–10 days later *6 months to 12 years post-surgery*	NHLBI-Fair
LYMQOL-Breast					
Riches 2020 [59] *N* = *40, retest n* = *25*	English	QOL *BLE assessment methods*	BLE: 59.98 ± 10.58 years All: 61.1 ± 9.6 years (29–80)	Baseline and 5–10 days later *6 months to 12 years post-surgery*	NHLBI-Fair
EORTC-BR23 (105 translations)					
Kilbreath 2020 [54] *N* = *89*	English *BLE* > *3 months*	QOL *RCT: Intervention-exercise*	Control: 59.5 ± 8.0 years Exercise: 53.7 ± 10.4 years	Baseline and 12 weeks	ROB-Low
Riches 2020 [59] *N* = *40, retest n* = *25*	English	QOL *BLE assessment methods*	BLE: 59.98 ± 10.58 years All: 61.1 ± 9.6 years (29–80)	Baseline and 5–10 days later *6 months to 12 years post-surgery*	NHLBI-Fair
Adriaenssens 2012 [36] *N* = *20*	NR	QOL *RT side effects and BLE diagnosis*	58.9 ± 12.1 years	Pre-surgery and post RT	NHLBI-Fair
Jankowska-Polanska 2017 [43] *N* = 50 (*150)	NR	QOL *Surgery effects/cosmesis*	BCS: 53.96 ± 8.54 (37–69 years)	Single measurement *20%* > *1 year, 24% 1–2 years, 56% over 2 years post-surgery*	NHLBI-Poor
Akca 2014 [44] *N* = 27 (*250)	Turkish	QOL *Surgery side effects/QOL*	Total sample: 47.4 ± 6.4 (28–55 years)	Single measurement	NHLBI-Poor
Adriaenssens 2012 [32] *N* = *131*	NR	QOL *RT side effects*	60.2 ± 10.4	Single measurement *Varied time post-surgery*	NHLBI-Fair
Eldridge-Hindy 2020 [30] *N* = *148*	English	QOL *RT side effects*	59 years (30–81)	Pre RT, End RT, 1, 6 months, 1, 2 and 3 years *Med 39.3 (range 6–94) months post RT*	NHLBI-Fair
De Oliveira-Junior 2021 [47] *N* = *300 (72 reconstruction)*	Brazilian Portuguese	QOL *Surgical outcomes*	59.8 (95%CI 58.6–60.98) (range 32.8–87.5) years	Single measurement *7.14 (95%CI 6.6–7.68) years post-surgery*	NHLBI-Good
Brandini da Silva 2019 [65] *N* = *300, n* = *50 (retest)*	Brazilian Portuguese	QOL *Assessment of BCTOS*	58.8 (25.6–87.5) years	Baseline and retest 21 to 30 days later *7.4 years (1.2–20.6) post first medical appointment*	NHLBI-Fair
Pukancsik 2017 [41] *N* = *200*	NR	QOL *Surgical side effects/cosmesis*	56 (32–70) years	Pre-surgery, 4 weeks post-surgery, 12 months post-surgery and post RT	NHLBI-Poor
Feisst 2019[66] *N* = *204*	German	QOL *Validation of BCTOS-12*	Med 57 (30–82) years	Single measurement *1–4 weeks post-surgery*	NHLBI-Fair
Struik 2018 [70] *N* = *101*	Dutch	QOL *Dutch translation of BCTOS-13*	61 (39–86) years	Single measurement, minimum 2–3 months post-surgery and post RT *14.6 (5–29) months post-surgery*	NHLBI-Fair
Heil 2011 [42] *N* = *199 (138 at f/up)*	German	QOL *Side effects*	58 years old ± 9.3 (95%CI 43–74)	7 days and 1 year post surgery *3–9 months post RT (median 7 months)*	NHLBI-Fair
Heil 2010 [69] *N* = *189*	German	QOL *Assessment of tool-German*	57 years	Single measurement *Mean 7.31 days post-surgery*	NHLBI-Poor
Hennigs 2018 [67] *N* = *871*	German	QOL *BCTOS item reduction*	58 ± 12.3(27–87) years	Single measurement *Median 4 days post-surgery*	NHLBI-Fair
BCTOS -22					
Weng 2021 [46] *N* = *287*	English	QOL *RCT-RT side effects*	CF-WBI: Med 60 (IQ: 54 –66) HF-WBI: Med 60 (IQ: 54 –66)	Pre-RT (*within 12 weeks of surgery*) and 6 months, 1,2,3,4,5 years post RT *Med f/up 48.3(IQR 42.3–49.6 months)*	ROB-Low
De Oliveira-Junior 2021 [47] *N* = *300*	Brazilian-Portuguese	QOL/Cosmesis *Surgical outcomes*	59.8 (95%CI 58.6–60.98) (32.8–87.5) years	Single measurement *7.14 (95%CI 6.6–7.68) years post-surgery*	NHLBI-Good
Chapman 2020 [40] *N* = *77*	English	QOL *RCT – RT side effects*	NR	Post-surgery/RT *3 years post RT*	ROB-Some concerns
Brandini da Silva 2019 [65] *N* = *300, N* = *50 (retest)*	Brazilian-Portuguese	QOL/Cosmesis *Reliability, translation*	58.8 (25.6–87.5) years	Baseline & retest 21 to 30 days later *7.4 years (1.2–20.6) post first medical appointment*	NHLBI-Fair
Jethwa 2018 [48] *N* = *131*	English	QOL/Cosmesis *RT side effects*	APBI: 69.3 ± 8.5 years WBI: 65.1 ± 10.8 years	Single measurement *Med 13.3 months post RT*	NHLBI-Poor
Teichman 2018 [49] *N* = *129*	English	QOL *RT side effects*	PBPT: 65 (5394) med 72.5 years WBI: 63.32 (46–86) med 70 years	Single measurement *PBPT, Mean* = *7.44 years; WBI, Mean* = *6.23 years post diagnosis*	NHLBI-Good
Vieira 2018 [68] *N* = *10 (v5), n* = *6 (v6)*	Brazilian Portuguese	QOL *Translation*	V5:57.9 ± 9.5, 42.2 ± 36.7 years V6:59.9 ± 10.6, 44.9 ± 43 years	Single measurement	NHLBI-Poor
Pukancsik 2017 [41] *N* = *200*	NR	QOL/Cosmesis *Surgical side effects/cosmesis*	56 (32–70) years	Pre-surgery, 4 weeks post-surgery, 12 months post-surgery and post RT	NHLBI-Poor
Ojala 2016 [50] *N* = *379*	Finnish/Swedish	QOL *Translation, surgical outcomes*	62 (36–92) years	Single measurement *3 years post-surgery*	NHLBI-Poor
Tian 2013 [45] *N* = 152 (*333)	English	QOL *Side effects*	62.4 ± 10.7 (55–77) years	Single measurement, at least 12 months post-surgery. *Mean 46 months post-surgery (12–136 months)*	NHLBI-Good
Heil 2010 [69] *N* = *189*	German	QOL/Cosmesis *Assessment of tool, translation*	Mean 57 years	Single measurement *Median 4 days, mean 7.31 days post-surgery*	NHLBI-Poor
Krishnan 2001 [38] *N* = *54*	English	QOL/Cosmesis *Cosmetic and functional status after (BCT) and relation to QOL*	64.34 ± 10.9 (41–83)	Single measurement *76.2* ± *48.33 months (9–216 months) post diagnosis*	NHLBI-Poor
Stanton 2001 [71] *N* = *184*	English	QOL *Validation of BCTOS-22*	61.62 ± 11.83 (28–85)	Single measurement *Mean 73.61 months post diagnosis* ± *51.45 (3–216 months)*	NHLBI-Fair
BCTOS-18 (Oedema subscale excluded)					
Eldridge-Hindy 2020 [30] *N* = *148*	English	QOL/Cosmesis *RT side effects*	59 years (30–81)	Pre RT, End of RT, 1, 6 months, 1, 2 and 3 years *Med f/up 39.3 (5.9–93.7 months) post RT*	NHLBI-Fair
Heil 2011 [42] *N* = *199 (138 at f/up)*	German	QOL/Cosmesis *Side effects*	58 years old (SD 9.3; 95% CI 43–74)	7 days and 1 year post surgery *3–9 months post RT (median 7 months)*	NHLBI-Fair
Swanick 2016 [39] *N* = *287*	English	QOL/Cosmesis *RT side effects/cosmesis*	CF-WBI median 60 (42–77) years HF-WBI median 60 (41– 81) years	Baseline, 6 months, 1, 2, 3 years post RT *Med f/up 24.7 months (IQR 13.3–36.3)*	ROB-Some concerns
BCTOS-12					
Feisst 2019[66] *N* = *204*	German	QOL/Cosmesis *Validation of BCTOS-12*	med 57 (30–82) years	Single measurement *1–4 weeks post-surgery*	NHLBI-Fair
Hennigs 2018 [67] *N* = *871*	English	QOL/Cosmesis *BCTOS item reduction*	58 ± 12.3(27–87) years	Single measurement *Median 4 days post-surgery*	NHLBI-Fair
BCTOS-13					
Struik 2018 [70] *N* = *101*	Dutch	QOL/Cosmesis *Shortened BCTOS & translation*	61 (39–86) years	Single measurement, 2–3 months post-surgery and RT *14.6 (5–29) months post-surgery*	NHLBI-Fair
Breast Symptom Scale					
Adriaenssens 2012 [32] *N* = *131*	NR	Symptom experience *RT side effects*	60.2 ± 10.4	Single measurement *Varied time post-surgery*	NHLBI-Fair
Breast Cosmesis Questionnaire					
Ashforth 2011 [55] *N* = *4*	English	Breast and skin density, skin appearance, swelling, pain *Int-JoViPitPak, SLD, compression*	39–55 years	Baseline, 2 weeks, 5 weeks of intervention	NHLBI-Poor
Modified DASH					
Kerrigan 2021 [72] *N* = *30*	English	Symptoms/function *BLE assessment*	Range ~ > 40 to > 70 years	Single measurement *6–24 months post-surgery*	NHLBI-Fair
Patient Rating Scales					
Ashforth 2011 [55] *N* = *4*	VAS	Breast pain *Int-JoViPitPak, SLD, compression*	39–55 years	Baseline, 2 weeks, 5 weeks of intervention	NHLBI-Poor
Johansson 2020 [51] *N* = *56*	VAS	Heaviness, pain, tightness *RCT: Int-compression*	Comp = 61.9 ± 7.6 years No comp = 61.3 ± 9.6 years	3 months post RT and 9 months	ROB-Some concerns
Collins 2018 [53] *N* = *14*	VAS	Heaviness/fullness, discomfort, redness *RCT: Int-Kinesiotape*	BLE Kinesio = 64.1 ± 5.9 years BLE Usual = 53.9 ± 10.4 years Overall = 59 years (34–74)	Baseline, end of treatment & 6 weeks post treatment *At least 4 weeks post radiotherapy*	ROB-Low
Jahr 2008 [56] *N* = *21*	VAS (pain) and 11-point scale (swelling)	Swelling, pain *RCT-interventions MLD* ± *deep oscillation*	Treatment: 56.6 (41– 65) years Control: 62.0 (42–71) years Total sample: 59.2 (41–71) years	Three measurements: baseline, 4 weeks (end of intervention), 8 weeks post intervention	ROB-Some concerns
Degnim 2012 [31] *N* = *124*	11-point scale	Heaviness, discomfort, redness, swelling *Assessment of BLE tool*	Med 56.5 (36–85) years	1, 3, 6, and 12 months post op *Median follow up 11 months (3–14 months)*	NHLBI-Fair
Heydon-White 2020 [73] *N* = *10*	Yes/no	Heaviness, discomfort *Assessment of tools*	54 ± 15 (36–81)	Single measurement	NHLBI-Fair
Jethwa 2018 [48] *N* = *131*	Linear analogue scale assessment	Symptom experience *RT side effects*	APBI: 69.3 ± 8.5 years WBI: 65.1 ± 10.8 years	Single measurement *Med 13.3 months post RT*	NHLBI-Poor
Ojala 2016 [50] *N* = *379*	Author developed questionnaire	QOL *BCTOS translation, surgical outcomes*	62 (36–92) years	Single measurement *3 years post-surgery*	NHLBI-Poor

Abbreviations: RT, radiotherapy; BLE, breast lymphoedema; Int, intervention; Med, median; AC, axillary clearance; MLD, manual lymphatic drainage; SLD, self lymphatic drainage; RCT, randomised controlled trial; NHLBI, National Heart, Lung and Blood Institute Quality Assessment Tool; ROB, Cochrane risk of bias tool version 2; BCT, breast conserving treatment; V, version; Comp, compression; EOT, end of treatment; CF-WBI, conventionally fractionated whole breast irradiation; HF-WBI, hypofractionated whole breast irradiation; WBI, whole breast irradiation; PBPT, proton beam radiation therapy; APBI, accelerated partial breast irradiation; ELIOT, intraoperative irradiation for early breast cancer; WBRT, whole breast radiation therapy; IORT, intraoperative radiation therapy; *whole sample including ineligible subjects for systematic review; QOL, quality of life

BrEQ, Breast Edema Questionnaire; BLYSS, Breast Lymphoedema Symptom Severity; LSIDS-T, Lymphedema Symptom Intensity and Distress Survey-Trunk; BLSQ, Breast Lymphoedema Symptom Questionnaire; LYMQOL-Breast, Lymphoedema Quality of Life tool-Breast; EORTC-QLQ BR23, European Organization for Research and Treatment of Cancer Breast Cancer-Specific Quality of Life Questionnaire; BCTOS, Breast Cancer Treatment Outcome Scale, QOL, quality of life; Modified DASH, Disabilities of the Arm, Shoulder and Hand; VAS, visual analogue scale

Measurement locations for the different tools and techniques varied and were either taken of the entire breast, quadrants or one or two selected locations on the breast. The entire breast was assessed for dermal backflow (ICG), volume (3D-SI, anthropomorphic, and MRI) and clinical rating scales. Ultrasound (dermal thickness), BIS, tissue resistance and TDC measures were performed both in breast quadrants [35, 36, 53, 57–60, 62, 73], two breast sites [21–24] or a single measurement site [52, 54, 55, 61, 72, 74]. TDC breast quadrant measures were also combined and reported as averages [19, 20, 51, 53, 59, 61] with the unaffected breast that was assessed to determine ratios. BIS measures were also reported as a ratio for the affected breast compared to the unaffected breast [54, 74].

### COSMIN summary: ClinROMs

The COSMIN Risk of Bias tool adapted for clinical measures (ClinROMs) [15] was completed for five clinical assessment tools [31, 57, 59–63], from eight studies that evaluated measurement properties. Evaluation of face validity, reliability and measurement error were conducted for all tools (Table 2). Criterion validity was not evaluated as there is no gold standard for measurement of breast lymphoedema. Measurement properties for dermal thickness measured with mammography as well as imaging signs and breast volume measured using anthropomorphic or MRI techniques were not reported in the studies meeting the inclusion criteria for this study. Only a single study presented measurement properties for clinician rating scales [31].

**Table 2:** Clinician Reported Outcome Measures (ClinROMs): measurement properties and COSMIN ratings

Assessment tool	Reference	Face validity	Reliability		Measurement error		
			Intra-rater	Inter-rater		Intra-rater	Inter-rater
Ultrasound (dermal thickness)	Dylke [57] (image measure)	+	ICC = 0.977 (0.7–0.93)	ICC = 0.96 (0.94–0.97)^a^			
				ICC = 0.85 (0.82–0.88)^b^			
				Cronbach’s α = 0.995 ^c^			
	Kilbreath [60] (image capture)	+	ICC = 0.84 (0.77–0.90)^d^			SEM = 0.122 mm, SEM% = 9.3%, SRD = 0.34mm^d^	
			ICC = 0.77 (0.66–0.84)^e^			SEM = 0.148 mm SEM% = 10.6%, SRD = 0.41mm^e^	
			ICC = 0.76 (0.65–0.84)^f^			SEM = 0.148 mm, SEM% = 10.6%, SRD = 0.41mm^f^	
			ICC = 0.66 (0.52–0.77)^g^			SEM = 0.141 mm, SEM% = 13.1%, SRD = 0.39mm^g^	
	Riches [59] (image measure)	+				BA mean diff = 0.008 (LOA − 0.857–0.873)	
	Overall rating (GRADE)	+ (NG)	** + (M)**	** + (M)**		? (M)	NR
TDC (local tissue water/percentage water content)	De Vrieze [61]	+	ICC = 0.95 (0.75–0.99)^h^	ICC = 0.0.90 (0.54–0.98)^h^		SEM = 2.2^ h^	SEM = 4.0^ h^
		+	ICC = 0.74 (0.00–0.94)^i^	ICC = 0.826 (0.30–0.96)^i^		SEM = 3.1^i^	SEM = 2.9^i^
		+	ICC = 0.78 (0.11–0.95)^j^	ICC = 0.86 (0.41–0.97)^j^			
	Riches [59]	+				BA mean diff = -0.23 (LOA = − 8.89–8.44)^j^	
	Overall rating (GRADE)	+ (NG)	+ (L)	+ (L)		? (L)	? (L)
Tonometry	Mosely [62]	?				CV = 1.29–3.25%	
	Riches [59]	- (^0^H)					
	Overall rating (GRADE)	- (NG)	NR	NR		? (VL)	NR
3D-SI (volume)	Leusink [63]	+				CV = 3.3%	
	Overall rating (GRADE)	+ (NG)	NR	NR		? (VL)	NR
BIS	Moseley [62]	+				CV = 0.29–0.86%	
	Overall rating (GRADE)	+ (NG)	NR	NR		? (VL)	NR
Clinician rating scale	Degnim [31] 2012	+	*K* = 0.76 (surgeon)/0.75 (LO therapist)				
	Overall rating (GRADE)	+ (NG)	? – not wK (L)	NR		NR	NR
Pitting	DeVrieze [61]	+	*K* = 0.36 (SE = 0.37)	*K* = − 0.102 (SE = 0.14)			
	Overall Rating (GRADE)	+ (NG)	- (L)	- (L)		NR	NR

Cut off values ICC: < 0.4 weak; 0.4–0.75 moderate; 0.75–0.9 strong; > 0.9 very strong (McDowell 1996) *Significant: *p* value < 0.05. Cut off values Cronbach alpha coefficients: < 0.5 unacceptable; 0.5– 0.6 weak; 0.6–0.7 acceptable; 0.7–0.9 good; > 0.9 excellent (Bland and Altman 1997; McDowell 1996)

BIS, bioimpedance spectroscopy; TDC, tissue dielectric constant; 3D-SI, three-dimensional surface imagery; SEM, standard error of measurement; SRD, smallest real difference; *wK*, weighted kappa; BA, Bland Altman; LOA, limits of agreement; diff, difference; *K*, kappa; CV, coefficient of variation; ^0^H, null hypothesis confirmed

COSMIN Overall ratings: + sufficient evidence;—insufficient evidence; ? indeterminate evidence; NR, not reported

GRADE: quality of evidence based on modified GRADE (risk of bias, inconsistency, imprecision (sample size) and indirectness [17]): H, high; M, moderate; L, low; VL, very low; NG, not graded. Bold denotes measurement criteria that has sufficient rating and a GRADE of moderate to high quality of evidence

^a^Assessor 1, ^ba^ssessor 2, ^c^interimage reliability, ^d^superior quadrant, ^e^inferior quadrant, ^f^ medial quadrant, ^g^lateral quadrant, ^h^affected breast, ^i^unaffected breast, ^j^percentage water content (PWC) ratio

Face validity was evaluated as *sufficient* for dermal thickness measurement using ultrasound [57, 59, 60], local tissue water measured with TDC [59, 61], breast volume measurement using 3D-SI [63], extracellular fluid measured with BIS [62], tissue resistance measured with pitting [61] and clinician rating scales of breast lymphoedema signs [31]. Tonometry face validity was evaluated as being *indeterminate* [59, 62] and having *insufficient* structural validity as this tool could not detect a difference between affected and unaffected breasts or lymphoedematous and non-lymphoedematous breasts [59]. Structural validity was not described for any other ClinROMs.

Reliability was rated as *sufficient* for measurement of dermal thickness for both the image capture [60] and image measurement [57, 59] using ultrasound, with a GRADE rating of *moderate* quality of evidence, due, in part, to low combined sample size (< 100) of studies that investigated it. Reliability was also evaluated as *sufficient* for TDC measuring percentage water content (PWC) ratio (affected:unaffected breasts) [59, 61]; however, it received a GRADE rating of l*ow* quality evidence due to imprecision (combined sample size for two studies < 50). Reliability of a clinician rating scale was *indeterminate* from a single study with GRADE rating downgraded to *low* quality due to risk of bias [31]. Pitting test reliability was rated as *insufficient* based on results from a single study with a GRADE of *low* quality due to small sample size (< 50) [59]. Reliability results were not available for tonometry, BIS, ICG or breast volume measurement.

Measurement error for all assessment tools was graded as *indeterminate* as minimally important change (MIC) has not been defined for any breast lymphoedema tools. Both dermal thickness assessed by ultrasound [57, 59, 60] and TDC [59, 61] had values for standard error of measurement and limits of agreement to allow some interpretation of results, with quality of evidence for measurement error graded as *moderate* for dermal thickness assessed by ultrasound and *low* for TDC, both of which were downgraded for the same reasons described for reliability respectively. Coefficient of variation was reported for tonometry [62], BIS [62] and breast volume measured by 3D-SI [63] with the quality of evidence for these tools graded as *very low*. There was no measurement error information for pitting test [59, 61] or clinician rating scales [31].

Based on the information provided, dermal thickness measurement assessed by ultrasound is *recommended* (Category A) for the assessment of breast lymphoedema as it has both have *sufficient* face validity and evidence for *sufficient* reliability with moderate quality of evidence. The other assessment tools, including TDC, BIS, tonometry, 3D-SI, clinician rating scales and the pitting test, are categorised as *promising* (Category B) as they do not have sufficient evidence for reliability, and the studies are of *low* or *very low* quality. No tools were categorised as insufficient (Category C).

### Characteristics of PROMs

Patient-reported breast lymphoedema symptoms severity and/or intensity were quantified in 14 studies with seven using questionnaires (Breast Lymphoedema Symptom Severity (BLYSS), Breast Edema Questionnaire (BrEQ), Disabilities of the Arm, Shoulder and Hand (Modified DASH), BSQ-Breast Symptom Questionnaire (BSQ), Lymphedema Symptom Intensity and Distress Survey-Trunk (LSIDS-T), Breast Cosmesis Questionnaire (BCQ), Breast Lymphoedema Symptom Questionnaire (BLSQ) and Breast Symptom Scale (BSS) [32, 54, 55, 58, 59, 64, 72]), six using visual analogue scales (VAS) [31, 48, 51, 53, 55, 56] and one using yes/no response options [73] (Table 1). Further, five questionnaires, European Organization for Research and Treatment of Cancer Breast Cancer-Specific Quality of Life Questionnaire (EORTC-QLQ BR23), Breast Cancer Treatment Outcome Scale-22, -13 and -12 (BCTOS-22, BCTOS-13, BCTOS-12) and Lymphoedema Quality of Life tool-Breast (LYMQOL-Breast), were used to quantify quality of life (QOL) as related to breast lymphoedema or associated symptoms in 24 studies. The EORTC QLQ BR23 was partly completed in three studies [41, 54, 70], using only the breast symptoms subscale with or without the arm symptom subscale, which measured symptoms rather than QOL, per se. The BCOTS-22, BCOTS-13 and BCOTS-12 had conflicting reporting of the construct it measured, with some studies reporting the subscales separately and used the subscales as a measure of cosmesis, pain and/or function [30, 38–42, 45, 46, 49, 50, 66, 67, 69–71], while others combined the subscales to measure QOL [47, 48, 65, 68].

### COSMIN-PROM

COSMIN for PROM [16] was used to evaluate nine PROMs from eleven papers [38, 58, 59, 64–71] meeting the inclusion criteria for this systematic review as well as two additional original validation papers in mixed breast cancer populations [75, 76] (Table 3).

**Table 3:** Patient Reported Outcome Measure (PROM) COSMIN ratings

Questionnaire	Content validity	Structural validity	Internal consistency	Reliability	Measurement error	Construct validity	Responsiveness
BrEQ [58]	** + (M)**	NR	? (L)	+ (VL)	NR	- (VL)	NR
BLYSS [64]	** + (M)**	NR	NR	+ (VL)	NR	? (VL)	NR
BCTOS-22 [38, 65, 68, 69, 71]	? (VL)	- (H)	** + (H)**	+ (VL)	NR	? (M)	- (VL)
BCTOS-12 [66, 67]	? (VL)	? (H)	** + (H)**	NR	NR	** + (H)**	NR
BCTOS-13 [70]	? (VL)	? (H)	? (H)	+ (L)	NR	? (L)	NR
BLSQ [59]	** + (M)**	NR	NR	+ (VL)	NR	** + (M)**	NR
LYMQOL-Breast ([59]	** + (M)**	NR	? (M)	- (VL)	? (L)	** + (M)**	NR
EORTC-BR23 [76]**	+ (L)	NR	? (L)	? (VL)	NR	+ (L)	+ (L)^a^
LSIDS-T [75]**	? (VL)	? (VL)	? (M)	NR	NR	+ (VL)	NR

BREQ, Breast Edema Questionnaire; BLYSS, Breast Lymphoedema Symptom Severity; BCTOS, Breast Cancer Treatment Outcome Scale; BLSQ, Breast Lymphoedema Symptom Questionnaire; LYMQOL-Breast, Lymphoedema Quality of Life tool-Breast; EORTC–QLQ BR23, European Organization for Research and Treatment of Cancer Breast Cancer-Specific Quality of Life Questionnaire; LSIDS-T, Lymphedema Symptom Intensity and Distress Survey-Trunk

^**^Indirect mixed breast cancer population; ^a^Spanish and Dutch versions only

COSMIN ratings, + ; sufficient rating, –; insufficient rating, ?; indeterminate rating, ()

Grading of overall quality of evidence based on modified GRADE approach; H, high; M, moderate; L, low; VL, very low; NR, not reported. Bold denotes measurement criteria that has sufficient rating and a GRADE of moderate to high

All included PROMs were evaluated as having *adequate* face validity; however, all PROM development studies lacked the detail required by the COSMIN methodology to score above a rating of *doubtful* quality. The BLYSS, BrEQ and EORTC-BR23 received a *doubtful* rating for quality of PROM design and the pilot study and a *doubtful* rating overall for PROM development. These three questionnaires consulted patients for concept elicitation using a qualitative approach but lacked detail on the interview and analysis process. Authors for the BSLQ and LYMQOL-Breast involved patients using quantitative methods for concept elicitation and to confirm comprehensibility and comprehensiveness but only performed this with a small sample of women (*n* = 20) resulting in an *inadequate* quality rating for PROM development.

The BCTOS-22/12/13 and LSIDS-T were all rated as *inadequate* for PROM design as they did not involve patients, either relying on literature review and experience of the authors (BCTOS), or only involving professionals in PROM design and pilot testing (LSIDS-T). A single exception was the pilot study testing the BCTOS-13 [70]. This study involved patients to rate comprehensibility and relevance resulting in a *doubtful* rating for quality for this pilot study.

The content validity studies for the nine PROMs similarly only achieved a maximum rating of *doubtful* quality. The BLYSS, LYMQOL-Breast, BLSQ, EORTC-BR23 and BrEQ were all rated as *doubtful* quality for relevance, comprehensiveness and comprehensibility due to lack of detail on the conduct and analysis of patient or professional interviews (BLYSS/EORTC-BR23/BrEQ) or only surveys being used (BLSQ, LYMQOL-Breast). The original studies for BCTOS were rated as *inadequate* quality for content validity. However, the German [69] and Brazilian-Portuguese [68] translations of BCTOS-22 did ask patients regarding comprehensibility but was rated as *doubtful* quality, due to limited information on analysis of this process. LSIDS-T content validity was also rated as *inadequate* due to lack of patient involvement in the content validity study.

Overall, BLYSS had *sufficient* quality of evidence with *moderate* grade evidence for content validity. The BrEQ, BLSQ and LYMQOL-Breast were also rated as *sufficient* with *moderate* GRADE evidence, with downgrading due to either lack of input from professionals (BrEQ) or use of quantitative methods in only a small sample for patient feedback (BLSQ/LYMQOL). The EORTC-BR23 had *sufficient* quality of evidence with *low* GRADE evidence due to indirectness of the sample used. LSIDS-T and BCTOS were both rated as *indeterminate* with *very low* GRADE evidence for content validity.

Six of the nine measurement properties were reported for the included PROMs (Table 3). Construct validity was evaluated for all questionnaires with a *sufficient* rating for six questionnaires (BrEQ, BCTOS-12, BLSQ, LYMQOL-Breast, EORTC QLQ BR23 and LSIDS-T). Internal consistency was evaluated for six questionnaires (BrEQ, BCTOS-22, BCTOS-12, BCTOS-13, LYMQOL-Breast, EORTC-BR23, LSIDS-T) with two measures receiving a *sufficient* rating (BCTOS-22, BCTOS-12) with *high* GRADE evidence. Reliability was evaluated for seven questionnaires (BrEQ, BLYSS, BCTOS-22 [Brazilian-Portuguese], BCTOS-13, BLSQ, LYMQOL-Breast, EORTC-BR23), with five achieving a *sufficient* rating (BrEQ, BLYSS, BCTOS-22, BCTOS-13, BLSQ), but the GRADE was *low* or *very low* for all. Structural validity was evaluated for four questionnaires (BCTOS-22, BCTOS-13, BCTOS-12, LSIDS-T), but no measure achieved a sufficient rating for this measurement property. The BCTOS-22 had an *insufficient* rating with *high* GRADE evidence. Responsiveness was only available for two questionnaires (BCTOS-22 and EORTC-BR23 [Spanish and Dutch versions]), with the EORTC-BR23 [Spanish and Dutch versions] achieving a *sufficient* rating with low GRADE evidence, and the BCTOS-22 rated as *insufficient* with *very low* GRADE evidence. Measurement error was presented for just one questionnaire (LYMQOL-Breast) and was rated as *indeterminate* with *low* GRADE evidence. Cross-cultural validity, criterion validity and measurement invariance were not presented for any questionnaires. There was no gold standard to assess criterion validity for PROMs.

### Quality assessment

Seven randomised controlled trials were included and assessed using the RoB 2 tool [18] (Table 1) [39, 40, 46, 51, 53, 54, 56]. Three RCTs had low overall risk of bias [46, 53, 54], and four were rated as having some concerns [39, 40, 51, 56]. Quality assessment for the 49 non-randomised studies [19–38, 41–45, 47–50, 52, 55, 57–74] was completed using the NHLBI quality assessment tool (URL: www.nhlbi.nih.gov/health-topics/study-quality-assessment-tools [accessed 26 October 2020]). Seven studies were rated as good quality [19, 45, 47, 49, 58, 60, 74], 25 rated as fair [20–24, 26, 27, 30–32, 35, 36, 42, 57, 59, 61, 63–67, 70–73] and 17 as poor [25, 28, 29, 33, 34, 37, 38, 41, 43, 44, 48, 50, 52, 55, 62, 68, 69] (Table 1).

## Discussion

The signs and symptoms of breast lymphoedema were quantified using a variety of approaches, including 13 patient-reported questionnaires, eight patient-reported rating scales, seven types of physical measures, seven clinician rating scales and four imaging techniques. Dermal thickness measured with ultrasound is recommended for assessment of breast lymphoedema, but further studies are required to establish the MCID and responsiveness (the validity of a change score). A breast lymphoedema PROM, however, cannot be recommended at this time as the reported details for development and measurement properties were lacking for all questionnaires. Nevertheless, the symptom-based PROMs, BLYSS, BLSQ and BrEQ (Dutch) and the QOL PROM LYMQOL-Breast are promising, with sufficient content validity. However, all tools require additional appropriately powered studies with women with, or at risk of breast lymphoedema to improve the measurement property evidence.

To fully assess the impact of breast lymphoedema, more than one assessment tool is suggested [7, 54, 77]. Breast lymphoedema is complex, with no agreed upon definition of the condition and with the presence of oedema in the breast influenced by treatment factors including surgery, radiotherapy and chemotherapy [78]. Measurement of signs and symptoms of breast lymphoedema, including both clinician- and patient-reported outcomes, would provide a comprehensive assessment of the underlying changes occurring. Forty-six percent of the included studies in this systematic review assessed more than one measurement outcome to quantify breast lymphoedema with 14 reporting both patient-reported and clinician-reported outcomes [31, 36, 40, 41, 47, 51, 53–56, 58, 59, 72, 73]. Inclusion of both ClinROMs and PROMs can also highlight the discord between patient and clinician reported outcomes, such as has been found in arm lymphoedema [12, 79], For example, measurements of dermal thickness provided information on the secondary tissue changes that can occur within the oedematous breast, but this does not necessarily relate to symptoms experienced by women [60]. Furthermore, due to the lack of a gold standard to assess the tools, we are unable to determine which tool is the best. Therefore, use of multiple tools, including those tools with the best available measurement property evidence, are recommended.

The practicality and expense of tools to quantify breast lymphoedema is a consideration for clinical usefulness. Questionnaires are the least expensive option, but responsiveness has only been established in EORTC-BR23 in non-English speaking samples. Ultrasound is readily available in hospitals and imaging centres but may be less accessible in private clinics where lymphoedema therapists often treat patients with lymphoedema. Comparably, TDC is a small, portable tool that could prove useful in clinical settings, but cost may still be prohibitive for small clinics at approximately $6000 AUD for a unit. Unfortunately, two reliable approaches that are widely used for limb lymphoedema, volume measurement and BIS [12], do not currently have sufficient evidence for breast lymphoedema assessment.

This review highlighted the need for standardised assessment protocols for the ClinROMs as there was heterogeneity across many of the studies on the measurement locations on the breast, with some studies reporting individual quadrant results while others only reporting overall means/ratios. For example, findings for dermal thickness measured with ultrasound and TDC may have been influenced by the location at which the measurement was taken. In healthy breasts, dermal thickness is greater in the inferior and medial breast quadrants [57, 59]; similarly, TDC varied across location in healthy breasts as well as unaffected breasts [59, 73]. Other factors such as age and menopausal status [80] and scar tissue [81] may also impact on breast signs but have yet to be investigated in the context of women with breast lymphoedema. Inclusion of these data may become important in the future in interpreting the findings.

This review identified significant gaps for the measurement properties of breast lymphoedema tools. The COSMIN framework for determining ratings for measurement properties is very comprehensive and relies on studies thoroughly reporting the study design to avoid poor ratings. Nevertheless, overall, there was a lack of high-quality evidence of measurement properties for breast lymphoedema tools. Dermal thickness measured with ultrasound had the most evidence but still lacked evidence of measurement error due to no established MCID for these or any breast lymphoedema tools. Four questionnaires (BrEQ, BLYSS, BLSQ, LYMQOL-Breast) were promising but require further investigation and larger sample sizes to improve overall quality of evidence for their measurement properties and overall quality of evidence. It is only after those investigations can recommendations to be made about their usefulness in assessment of breast lymphoedema.

## Conclusion

The findings from this systematic review reveal that ultrasound has the best measurement properties, including information on measurement error, but MIC has not yet been established. Of the PROMS, BLYSS, BrEQ and BLSQ for symptom severity and LYMQOL-Breast for measurement of QOL are promising tools to assess women following conservative breast cancer treatment. Well-designed and reported studies on measurement properties for all tools are required to improve quality of evidence in this emerging area of assessment. Based on the current level of evidence, a combination of objective and subjective measurements is recommended to quantify the full manifestation of breast lymphoedema signs and symptoms.

## Systematic Review Registration

This systematic review was registered in PROSPERO (CRD42020183851).
